# Contrasting outcomes of genome reduction in mikrocytids and microsporidians

**DOI:** 10.1186/s12915-023-01635-w

**Published:** 2023-06-06

**Authors:** Vojtečh Žárský, Anna Karnkowska, Vittorio Boscaro, Morelia Trznadel, Thomas A. Whelan, Markus Hiltunen-Thorén, Ioana Onut-Brännström, Cathryn L. Abbott, Naomi M. Fast, Fabien Burki, Patrick J. Keeling

**Affiliations:** 1 Department of Botany, University of British Columbia, V6T 1Z4, Vancouver, 3529-6270 University Boulevard, BC Canada; 2 Institute of Evolutionary Biology, Faculty of Biology, University of Warsaw, 02-089 Warsaw, Poland; 3 Department of Organismal Biology, Uppsala University, Norbyv. 18D, 752 36 Uppsala, Sweden; 4 Department of Ecology, Environment and Plant Sciences, Stockholm University, SE-106 91, Stockholm, Sweden; 5 Department of Ecology and Genetics, Uppsala University, 752 36 Uppsala, Sweden; 6 Natural History Museum, University of Oslo, 0562 Oslo, Norway; 7 Pacific Biological Station, Fisheries and Oceans Canada, Nanaimo, BC V9T 6N7 Canada

**Keywords:** Genomics, Evolution, Parasite, Reduction, Anaerobic, Intron, Splicing, Metabolism, *Mikrocytos*, Microsporidians

## Abstract

**Background:**

Intracellular symbionts often undergo genome reduction, losing both coding and non-coding DNA in a process that ultimately produces small, gene-dense genomes with few genes. Among eukaryotes, an extreme example is found in microsporidians, which are anaerobic, obligate intracellular parasites related to fungi that have the smallest nuclear genomes known (except for the relic nucleomorphs of some secondary plastids). Mikrocytids are superficially similar to microsporidians: they are also small, reduced, obligate parasites; however, as they belong to a very different branch of the tree of eukaryotes, the rhizarians, such similarities must have evolved in parallel. Since little genomic data are available from mikrocytids, we assembled a draft genome of the type species, *Mikrocytos mackini*, and compared the genomic architecture and content of microsporidians and mikrocytids to identify common characteristics of reduction and possible convergent evolution.

**Results:**

At the coarsest level, the genome of *M. mackini* does not exhibit signs of extreme genome reduction; at 49.7 Mbp with 14,372 genes, the assembly is much larger and gene-rich than those of microsporidians. However, much of the genomic sequence and most (8075) of the protein-coding genes code for transposons, and may not contribute much of functional relevance to the parasite. Indeed, the energy and carbon metabolism of *M. mackini* share several similarities with those of microsporidians. Overall, the predicted proteome involved in cellular functions is quite reduced and gene sequences are extremely divergent. Microsporidians and mikrocytids also share highly reduced spliceosomes that have retained a strikingly similar subset of proteins despite having reduced independently. In contrast, the spliceosomal introns in mikrocytids are very different from those of microsporidians in that they are numerous, conserved in sequence, and constrained to an exceptionally narrow size range (all 16 or 17 nucleotides long) at the shortest extreme of known intron lengths.

**Conclusions:**

Nuclear genome reduction has taken place many times and has proceeded along different routes in different lineages. Mikrocytids show a mix of similarities and differences with other extreme cases, including uncoupling the actual size of a genome with its functional reduction.

**Supplementary Information:**

The online version contains supplementary material available at 10.1186/s12915-023-01635-w.

## Background

One of the most consistent trends observed in the evolution of intracellular parasites and symbionts more broadly is that obligate intracellular organisms undergo genome reduction [1–3]. Genome size, gene number, and even non-coding sequence length decrease in endosymbionts compared to their free-living ancestors, while sequence substitution rates often increase. Sometimes these changes can be drastic [4–6]. Alternatively attributed to adaptive streamlining of non-essential functions or neutral loss due to weakened selection [2, 7], there are few exceptions to this outcome. While genome reduction is mostly studied in specialized pathogenic or ancient mutualistic bacteria [8, 9], it occurs in a wide variety of contexts, and not just in prokaryotes but also in eukaryotes. A notable case is found in Microsporidia [10, 11], unicellular protists related to fungi with several unusual features [12, 13]. Microsporidians are obligate intracellular parasites that can be pathogenic in immunocompromised humans and cause widespread diseases in other animals, including economically important species such as bees and silkworms [14–16]. While microsporidian spores possess a complex infection mechanism, the cells are highly reduced in nearly every other way. Their metabolism is so limited that microsporidians have lost most or in some cases all ATP production pathways and steal ATP directly from their hosts [17–19]. Their mitochondria evolved into anaerobic “mitosomes” lacking a genome and seemingly having the only function of synthesizing Fe-S clusters [20]. Microsporidian nuclear genomes are typically also highly reduced and include the smallest nuclear genome known in any cell: 2.3 Mbp and 1800 protein-coding genes in *Encephalitozoon intestinalis* [4]. As models for nuclear genome reduction and compaction, microsporidian genome content, gene density, and introns have all been studied in some detail [4, 21, 22].

An interesting potential lineage to compare and contrast with microsporidians are the mikrocytids, a more recently discovered group of parasites of marine invertebrates currently comprising only a few described species [5, 23–25]. Mikrocytids belong to the understudied eukaryotic “supergroup” Rhizaria [5] and are therefore only distantly related to Microsporidia. However, the two lineages share a host-dependent, intracellular lifestyle and some convergent features including the reduction of mitochondria to mitosomes [5, 26]. The transcriptome of the mikrocytid *Mikrocytos mackini*, the causative agent of Denman Island disease in oysters [27], showed the fastest sequence substitution rate of any known eukaryote [5], suggesting once again a marked effect of endosymbiosis on molecular evolution.

While genome reduction is common to intracellular organisms, its extent, underlying mechanisms, and the order of events leading to it are not the same in different lineages, especially among eukaryotes. To examine some of the similarities and differences in the process, here we sequenced the genome of the mikrocytid *M. mackini* and compared its overall characteristics (as well as those of the recently reported genome of its closest known relative, *Paramikrocytos canceri* [26]) with the more thoroughly studied genomes of microsporidians.

## Results and discussion

### The *Mikrocytos* genome is large and gene-rich

*Mikrocytos mackini* is a tiny (< 5 μm), strictly intracellular parasite that cannot be cultured outside its host. To obtain as clean an assembly as possible in such circumstances, *M. mackini* cells were isolated from the tissue of the host, the Pacific oyster *Crassostrea gigas*, and libraries constructed from the inevitably low DNA yield. The final 49.7 Mbp assembly appears to be largely complete, albeit very fragmented (16,018 contigs; N50 = 4547 bp; Table 1), which is likely at least in part due to a high number of repetitive sequences (see below). The relative completeness of the assembly is evidenced by the high percentages of RNA-Seq reads mapping against the genome draft (96%) and of detected orthologs from a dataset [28] of 263 highly conserved eukaryotic genes (80%). Differences in metabolic gene sets were further inspected between the *M. mackini* transcriptome and genome, and only three genes were found in the former but not the latter: two were part of the assembly, but split across separate contigs, and one was entirely missing, although its predicted function was represented by other paralogs. Additionally, all rRNA, tRNA, and tRNA synthetase genes were present, as well as most ribosomal protein genes (75%). Low BUSCO scores (36%) have been recovered before for genomic data from protists belonging to undersampled groups [26, 29, 30] and are likely the consequence of sequence divergence and poor representation in reference databases. The *M. mackini* assembly is considerably larger than those obtained from other rhizarian parasites like *Plasmodiophora brassicae* (24 Mbp [31]) and *Paramikrocytos canceri* (13 Mbp [26]).

**Table 1:** Assembly statistics for genomes of mikrocytids (*Mikrocytos mackini* and *Paramikrocytos canceri*) and other available rhizarians

	***Mikrocytos mackini*** **(parasite)**	***Paramikrocytos canceri*** **(parasite)**	***Plasmodiophora brassicae*** **(parasite)**	***Bigelowiella natans*** **(free-living)**	***Reticulomyxa filosa*** **(free-living)**
Assembly size (Mbp)	49.7	12.7	24.0	94.7	101.9
No. contigs	16,018	3,113	165	302	50,809
N50	4547	6806	472,887	819,951	3609
Max. contig length (bp)	76,988	40,223	1,189,627	3,030,241	48,547
GC content	33.60%	30.06%	59.40%	44.90%	35.00%
No. of protein-coding genes	14,372	8201	9,913	22,320	40,160
Reference	This paper	[26]	[31]	[32]	[33]

Although the sample size is limited by the scarcity of data on Rhizaria overall, the genome reduction trend is confirmed in this eukaryotic supergroup, with much smaller genomes observed for parasitic than free-living organisms (Table 1). Its extent is however not as dramatic as in microsporidians, where genome sizes vary widely (2–50 Mbp) but are usually well below 10 Mbp [22]. The difference is more prominent in the number of protein-coding genes, uniformly low in microsporidians (2000–5000) and higher in mikrocytids (14,372 predicted putative genes in *M. mackini*, > 8000 in *P. canceri*). This cannot simply be attributed to a more recent origin of parasitism or slower progression of DNA loss, since *M. mackini* and *P. canceri* undoubtedly share an already parasitic common ancestor but have considerably different degrees of genome reduction, suggesting a more complex dynamic, possibly linked to the surprisingly high number of transposons in *M. mackini*.

### The *M. mackini* genome encodes abundant and diverse transposable elements

According to models developed in bacteria, genome reduction usually goes through an early, chaotic stage characterized by the uncontrolled spread of mobile elements (due to relaxed purifying selection), which in turn facilitates chromosome rearrangements and pseudogenization [34], and a later more stable stage involving loss of non-essential sequences (including mobile elements) and compaction [2]. There are exceptions to this rule [35], and the progression has not been established in eukaryotes. We did, however, observe a large number of mobile elements in the genome of *M. mackini*: 8075, or 56%, of the predicted protein-coding genes show signatures of transposon origin, as do many other regions of the genome (Table 2). About half of the predicted transposable elements (TE) could be assigned to known families, especially long terminal repeats, long interspersed nuclear elements, terminal inverted repeat-containing DNA transposons, and helitrons. The *P. canceri* genome assembly also encodes TEs, albeit to a much lower extent (Table 2). If this is an accurate reflection of both genomes, as seems to be the case, the rates of TE spread and/or loss in the two lineages must have been highly dynamic.

**Table 2:** Number and classification of transposable elements in mikrocytid genomes

		***M. mackini***	***P. canceri***
**Total**		**32,821**	**13,633**
**Retrotransposons**	**LTR**	4228	298
	**LINE**	1730	1148
**DNA transposons**	**TIR**	8228	1877
	**Helitrons**	792	0
**Unclassified**		17,843	10,310

*LTR* long terminal repeats, *LINE* long interspersed nuclear element, *TIR* terminal inverted repeat

**Fig. 1:**
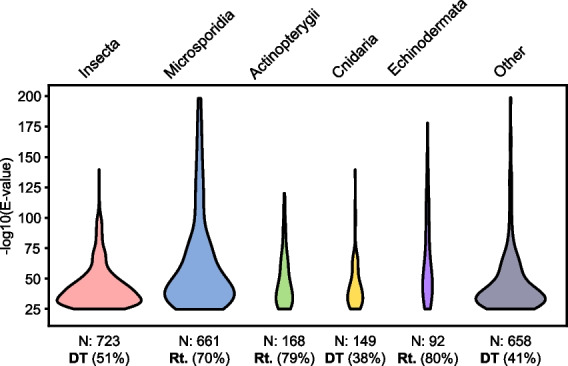
Sources of transposable elements in the genome of *M. mackini*. Each graph shows the distribution of BLAST hits against selected taxa of animals and protists. The number of reliable (*e*-value < 1e^−20^) hits shown for each target group is reported (N). The percentage of hits belonging to either DNA transposons (DT) or retrotransposons (Rt.), whichever is higher, is also shown

It is also possible that the *M. mackini* genome is the result of more recent “invasions” of transposons in an already reduced genome under weak purifying selection. This seems to be the case for many microsporidians, where species with larger genomes have more TEs than species with the most reduced genomes, which have few or none [36, 37]. Since the last common ancestor of extant microsporidians was already an intracellular parasite with a highly reduced gene content, this diversity is unlikely to reflect the ancestral state, but rather later TE invasions that produced secondary genome bloat. Further evidence in microsporidians comes from the sources of TEs, which were seemingly acquired from a variety of animals, probably reflecting host shifts over their evolutionary history [38, 39]. Similarly, TE sequences in *Mikrocytos* show relatively high similarities with homologs in, among others, ray-finned fishes, echinoderms, insects, cnidarians, and even microsporidians (the latter likely indicating an exchange between co-occurring parasites) (Fig. 1). More genomes from mikrocytids are required to confirm the observed pattern and exclude any influence from undetected contaminant sequences, which are always a possibility when working with intracellular organisms. However, the current data lend more support to a differential transposon acquisition rather than the unchecked multiplication of ancestral TEs scenario.

Interestingly, another strong correlation found in microsporidians is the presence of Argonaute and Dicer components of the RNAi machinery in all TE-rich genomes [39, 40], which is not the case in *M. mackini*, where orthologs of these genes could not be identified.

### Microcytids have many, extremely short introns of highly uniform length

**Fig. 2:**
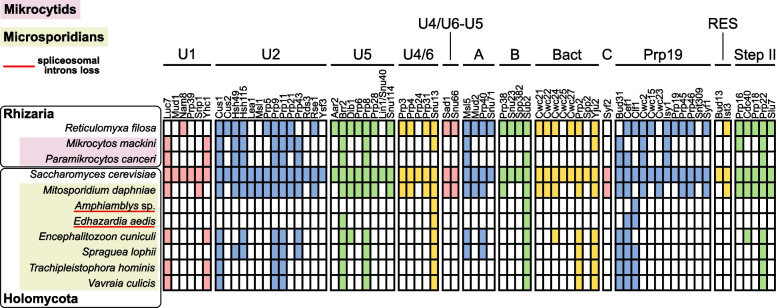
Spliceosome convergent reduction in mikrocytids and microsporidians. The table shows the presence/absence of spliceosomal protein components in the genomes of the two mikrocytids, selected microsporidians, and two non-reduced relatives for reference: the free-living *Reticulomyxa filosa* (Rhizaria) for mikrocytids and the yeast *Saccharomyces cerevisiae* (Holomycota) for microsporidians. Microsporidians that have completely lost spliceosomal introns are underlined in red

**Fig. 3:**
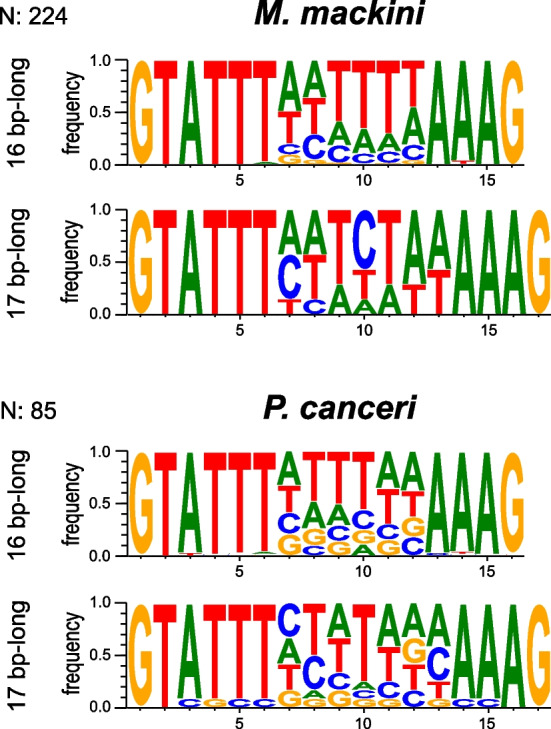
Conserved sequences of the extremely short spliceosomal introns detected in both mikrocytids, *M. mackini* (top) and *P. canceri* (bottom). N stands for the total number of spliceosomal introns found in each genome

In obligately symbiotic bacteria, the shortening of non-coding sequences during genome reduction generally means short intergenic regions. In intracellular eukaryotes, the trend can also extend to introns, either due to loss, length reduction, or both. Microsporidians generally have few introns that are relatively short and retain a higher-than-average sequence similarity to one another [18, 21, 41, 42], as well as a reduced spliceosomal machinery [41, 43, 44]. A few microsporidians have independently lost introns altogether [18, 45, 46]. The spliceosome in mikrocytids seems to be almost as small (only 17–19 proteins plus the U2, U4, and U6 snRNAs were identified), and there is a striking degree of overlap in the proteins that have been retained in the two groups, despite their independent spliceosome reduction (Fig. 2). In contrast to the intron-poor genomes of microsporidians, however, our annotation predicted 224 introns in 179 genes in the genome of *M. mackini*. These introns are incredibly small and very uniform in length: nearly all were 16 bp long (217/224), and the rest were 17 bp long (7/224). Comparing the genome to transcriptomic data showed that 16 bp introns spliced twice as frequently as 17 bp introns (63% vs. 37%, respectively). Moreover, all introns shared highly conserved sequences (Fig. 3). About half of the intron-containing *M. mackini* genes were functionally annotated, revealing that most are involved in essential functions related to gene expression (including DNA damage repair, RNA transcription, splicing, etc.) and cell-cycle regulation (Additional file 1: Fig. S1). No intron was found in metabolic enzyme or transporter genes. Examining the *P. canceri* assembly revealed that it too contains introns with these same characteristics (Fig. 3).

It is generally unclear why introns vary so much in length and number, from more than 90,000 in the ciliate *Paramecium* [47] to few or none in microsporidians, trypanosomes, and other protists [46, 48, 49]. The yeast *Saccharomyces cerevisiae* has relatively few (282) and long (~ 400 bp) introns, which play an important role in gene expression regulation [50, 51]. There is no strong evidence for the same function in microsporidians, despite some similarities in intron distribution and localization (as in yeasts, they are often found at the 5′ end of ribosomal protein genes). Another reduced genome rich in introns (more than 800 in approximately 300 genes) is found in the chlorarachniophyte nucleomorph, a remnant nucleus of secondary plastids derived from an ancient symbiosis with a green alga [52]. The nucleomorph genome is another example of extreme genome reduction in an intracellular symbiosis and, like those of mikrocytids, its introns are not only short, but also fall into a narrow size range: 18 to 21 bp in this case. The smaller and more narrowly constrained introns of mikrocytids are matched only by the 15–16 bp introns of heterotrich ciliates [53, 54], which, seemingly against the trend, are free-living organisms with very large cells, nuclei, and genomes.

Intron reduction is likely occurring in different systems for different reasons, so seeking a single unifying explanation may be fruitless. In yeasts, many introns are hypothesized to be maintained for functional reasons [55], but alternative neutral explanations are also possible. For instance, like other non-coding sequences, introns in endosymbionts might simply gradually shrink in size due to genome erosion, where reduced DNA repair mechanisms lead to a bias for deletions over insertions. This could presumably continue until a functional threshold is hit, below which the introns might be too short to be efficiently spliced and further gradual reductions would be strongly deleterious [54]. This threshold could be slightly different in systems evolving independently, for instance because introns in organisms with lower intron densities and reduced spliceosomes also tend to evolve greater dependence on sequence conservation for spliceosomal recognition and base-pairing with the snRNAs [56, 57]—the longer the recognition sequence, the longer the minimal intron size. A balance between this threshold and the deletion bias would lead introns to fall into a narrower and narrower size range, bounded on one side by their functional minimal length and eroded on the other by the strength of the deletion-bias.

### Divergent genes and reduced metabolism of *M. mackini*

**Fig. 4:**
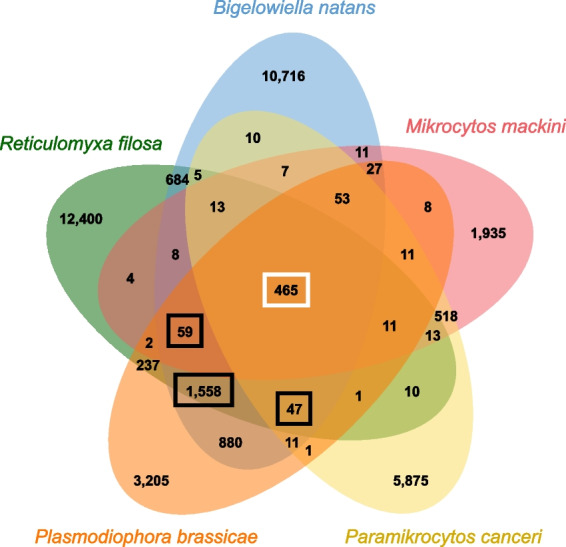
Shared orthogroups in rhizarian genomes. The Venn diagram shows the numbers of orthologous groups of genes inferred by Orthofinder in the five available rhizarian genomes. Highlighted with a white rectangle are the orthogroups shared by all rhizarians, including mikrocytids (465). Highlighted in black rectangles are additional orthogroups shared by all non-mikrocytid rhizarians (for a total of 2129)

Relatively few protein-coding genes unrelated to transposable elements (2072, or 33%, out of 6297) in the genome draft of *M. mackini* could be functionally annotated. While this is in part due to the paucity of data on close relatives of mikrocytids, an even larger effect is probably played by the sequence divergence characterizing this protist [5]. Indicative of this is the fact that rhizarians share only 465 gene orthogroups if mikrocytids are included, but 2129 if they are not (Fig. 4).

As in other parasites, many metabolic pathways that are considered essential in free-living eukaryotes are absent from *M. mackini*. Significantly, both *M. mackini* and *P. canceri* share a rare trait with microsporidians: the absence of the ATP synthase complex, as well as associated pathways like the carboxylic acid cycle and beta oxidation. Genes for a full glycolysis pathway are present in the *M. mackini* genome, suggesting that some ATP can be produced by substrate-level phosphorylation. Another parallel with microsporidians [58] is the preservation of trehalose metabolism genes in an otherwise depleted carbon metabolism (Additional file 2: Fig. S2). Trehalose plays a role in carbohydrate storage in many invertebrates [59], and this together with its retention in *M. mackini* indicate that this compound might be important to the interactions between microcytids and their hosts. We additionally detected a putative trehalase gene with a signal peptide, suggesting it is secreted from the parasite cell, possibly to modulate and redirect the flow of carbohydrates in the host’s cytoplasm. A similar use of trehalase has been predicted in microsporidians [60].

**Fig. 5:**
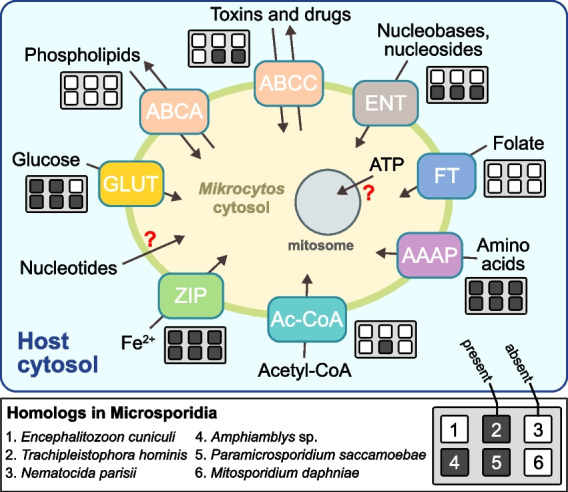
Predicted types of transporters identified in the *M. mackini* genome and differences with the microsporidian sets. The red question marks stand for metabolite exchanges that are supposed to happen, but for which no good candidate gene was detected. Next to each transporter predicted for *M. mackini*, a small plot shows which of the representative microsporidians have corresponding homologous genes. The set differs in many details between mikrocytids and microsporidians, as well as within microsporidians (as a further note, while microsporidians lack ABCA transporters, they possess the functionally related ABCG, which is missing in *Mikrocytos*). AAAP, amino acid/auxin permease; ABC, ATP-binding cassette transporter (types A and C); Ac-CoA, acetyl-CoA transporter; ENT, equilibrative nucleoside transporter; FT, folate transporter; GLUT, glucose transporter; ZIP, zinc/iron permease

Overall, gene content supports a metabolic convergence between microsporidians and mikrocytids to energy parasitism, or the direct acquisition of some or all of the parasite’s ATP from the host. This prediction is consistent with the close association to the host cell’s mitochondria that is observed both in mikrocytids [25, 61] and microsporidians [62, 63]. However, it should be noted that among the 61 transporter genes, representing 17 families, annotated in *M. mackini* (a more reduced repertoire than that of microsporidians [64]), we did not find a clear candidate ATP transporter (Fig. 5). The same was true for *P. canceri* [26]. The bacterial-derived nucleotide transporter (NTT) microsporidians use to import ATP [17] was not present in *M. mackini*, although we did identify the more common equilibrative nucleoside transporter (ENT). Also notably absent from mikrocytids are the mitochondria carrier family (MCF) genes, responsible for the transport of metabolites in mitochondria and mitosomes, which have also been replaced by bacterial transporters in some microsporidians [17, 64]. Considering how common horizontal gene transfers are, and inherent difficulties in transporter annotation, we cannot state that a particular transport function is missing in mikrocytids, but it seems likely that when it comes to transporters, these parasites often rely on different protein families than microsporidians to perform similar, key functions (Fig. 5).

## Conclusions

**Fig. 6:**
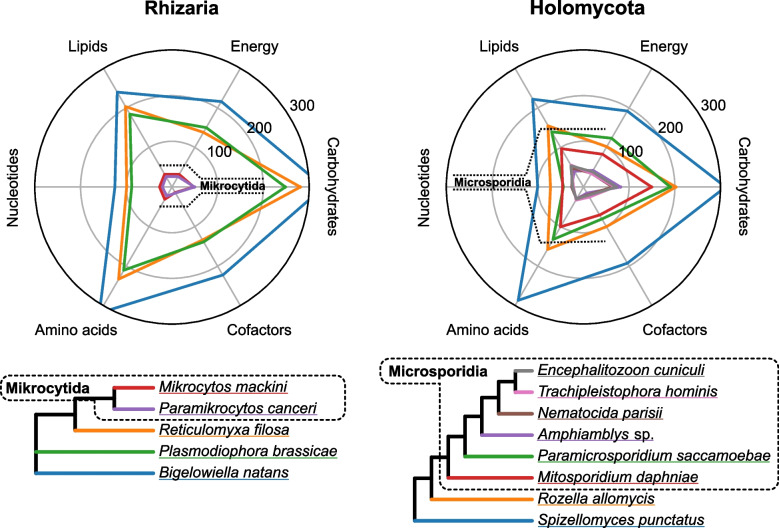
Convergent minimal metabolism of mikrocytids and the most reduced microsporidians. Plots are shown for all sequenced rhizarians including mikrocytids (left), and selected holomycotes (fungi and relatives, such as microsporidians and rozellids). Cladograms depict the phylogenetic relationships of the analyzed taxa. On each radial axis, representing a major KEGG metabolic category, the number of unique enzymes is plotted, showing a convergence of the most genome-reduced representatives of each group to similarly depleted enzyme sets

Superficially, microsporidians and mikrocytids have a lot in common: they are intracellular parasites of other eukaryotes with tiny cells, mitosomes, and peculiar genomic traits. In fact, we have shown here that these two lineages have also converged to a similar form of very reduced metabolism with shared, rare features (Fig. 6). However, microsporidians and mikrocytids provide very different examples of how the process of genome reduction can develop. *Mikrocytos mackini* is an unusual case study for extensive, multiple transposon invasions in the context of an otherwise reduced genome, as well as extreme intron length reduction without outright loss.

## Methods

### Cell isolation, library preparation, and sequencing

*Mikrocytos mackini* were collected from parasitic lesions on the adductor muscle tissues of wild *Crassostrea gigas* harvested from Deep Bay (Vancouver Island, British Columbia, Canada), then used to infect oysters in the lab in order to generate sufficient material for nucleic acid extractions. Parasites were concentrated and isolated from the lab-infected hosts as described in [5]. DNA was extracted with the DNeasy Blood & Tissue Kit (Qiagen) following the manufacturer’s protocol. About 4 μg of DNA was submitted to the Génome Québec sequencing center for library preparation and sequencing. TruSeq paired-end libraries were sequenced on the Illumina MiSeq (2 × 250 bp and 2 × 300 bp) and HiSeq (2 × 100 bp) platforms.

### Genomic and transcriptomic assemblies

Adaptor sequences were removed and low-quality sequences trimmed from genomic reads using fastq-mcf [65]. Host contaminant reads were identified through mapping against a *Crassostrea gigas* reference genome using Megablast as implemented in the BLAST + package [66], then removed (thresholds: > 90% identity and > 40% hit coverage). This first filter culled about 30% of the data. A preliminary assembly was built using Ray [67] and the contigs were aligned using BLAST against the NCBI nt database. Four potential *C. gigas* contigs were flagged and reads mapping to those contigs were removed. Remaining redundant reads were discarded using the normalize-by-median.py script of the khmer package [68].

Three assemblies were built using Ray (v.2.3.1), SPAdes (v.3.6.1) [69], and MIRA (an iterative assembler; three passes were used) [70]. The assemblies were first compared by mapping transcripts against each of them with gmap (v.2020-04-08) [71], which produced values of 91.9%, 92.7%, and 95.8% for the outputs of Ray, SPAdes, and MIRA, respectively. Then, ALE [72] was run to estimate likelihood values, with the MIRA assembly obtaining the highest score. The final genome draft was then created by selecting large contigs from the MIRA assembly (> 550 bp) and adding shorter contigs that did not have a match against the large contigs. A final decontamination step was performed by searching against the NCBI nt database using Megablast [73] and removing contigs matching *C. gigas*.

Transcriptomic reads from a previously reported study [5] were also re-assembled to examine genome completeness. Raw reads were trimmed using Trimmomatic [74] and assembled de novo using Trinity [75]. Common contaminants were detected using blobology [76] and the reads were filtered through mapping against database of identified contaminants with bwa [77]. De novo and genome-guided assemblies using only decontaminated reads were built again on Trinity, and a comprehensive set of transcripts was generated using the build_comprehensive_transcriptome.dbi script from the PASA pipeline (v.2) [78].

### Genome annotation

Preliminary gene predictions were performed using the PASA pipeline (v.2) to align transcripts to the assembly, Genemark [79], and Augustus (v.3.0.3) [80]. The final set of predicted genes was generated using EVM (v.r2012-06-25) [81] with inputs from all three other programs. BUSCO (v.5) [82] was run using the alveolata_odb10 dataset to obtain a completeness estimate.

Predicted protein-coding genes were annotated using eggNOG-mapper (v.2) [83] and Interproscan (v.5.50) [84]. The KEGG database of orthologs [85] was searched using HMMER [86] and served as the basis for the classification of enzymes and metabolic pathways. Orthologous protein-coding gene groups (orthogroups) were predicted for rhizarian genomes using OrthoFinder (v.2.5.2) [87] with default settings. The Venn diagram of shared orthogroups was created using OrthoVenn2 [88]. rRNA, tRNA, and snRNA genes were predicted using Infernal cmscan (v.1.1.3) [89] against the Rfam database (v.14) [90]. Metabolic graphs were built by counting the number of unique enzymes annotated in major KEGG Pathway Families (energy metabolism, carbohydrate metabolism, metabolism of cofactors and vitamins, amino acid metabolism, nucleotide metabolism, lipid metabolism), then plotting the numbers on radial axes using the polar plot projection as implemented in Matplotlib (v.3.7) [91].

Transposable elements in the *M. mackini* genome were detected and classified using RepeatModeler (v.2.0.3) [92] with the -LTRStruct option. TEs were then compared against the NCBI nt database using diamond blastx (v.2.0.7) [93] and best hits with *e*-values < 1e^−20^ (amino acid similarity values ranged from 74.8% to 21.4%; average: 35.2%) were collected and sorted by taxonomic group.

Putative introns were first pinpointed by mapping transcripts from *M. mackini* onto the genome draft using gmap with the –min-intron-length 10 option. RNA-Seq reads were also mapped against the genome using the splice-aware aligner TopHat (v.2.1.1) [94] with the same length restriction. Mapped reads were then used to assess the exon coverage, count intron-spanning reads, and estimate splicing efficiency of putative intron–exon boundaries. The conservation of intron sequences was visualized using WebLogo (v.3) [95]. The gene ontology enrichment analysis of genes with spliceosomal introns was performed using Ontologizer (v.2.1) [96] and visualized with GO-Figure! (v.1.0) [97]. Spliceosomal proteins were detected using reciprocal BLAST against the human and yeast proteomes and candidates were checked using the HHpred server [98]. Sm and Lsm proteins were not included in the analysis as they are very short and unreliably differentiated based on sequence similarity alone.

## Supplementary Information

**Additional file 1: Figure S1.** Gene Ontology enrichment analysis of genes with spliceosomal introns in the genome of *M. mackini* plotted in the GO semantic space using GO-Figure!. The colour scale represents the significance of the enrichmentand the size of the circles stands for the number of genes with spliceosomal introns with that particular annotation. Where available, functional categories are shown in the legend.

**Additional file 2: Figure S2.** Predicted carbohydrate metabolism of *M. mackini*. The presence of a putative lactate dehydrogenaseenzyme has been deduced from the comparison with LDH / malate dehydrogenasehomologs.

## Data Availability

The datasets generated and analyzed during the current study are available in the GenBank database at the following link: https://www.ncbi.nlm.nih.gov/bioproject/PRJNA940158 [99].

## Abbreviations

bp: Base pairs
ENT: Equilibrative nucleoside transporter
MCF: Mitochondria carrier family
NTT: Nucleotide transporter
TE: Transposable elements
