# Supplementary figures and images for: Contrasting outcomes of genome reduction in mikrocytids and microsporidians
Source: BMC Biol. 2023 Jun 6;21:137. doi: 10.1186/s12915-023-01635-w (PMC10245619; doi:10.1186/s12915-023-01635-w)

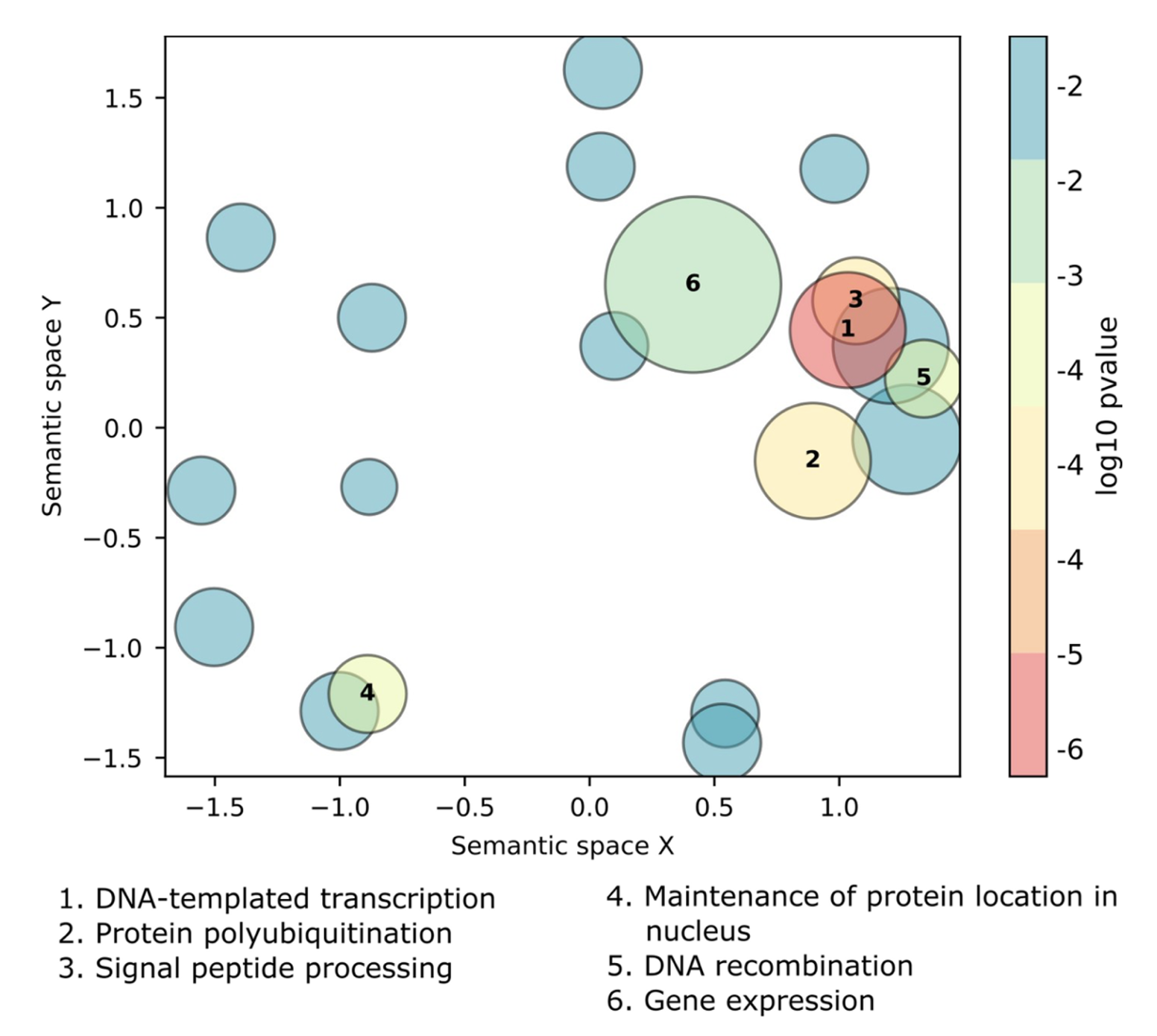

Supplement: Supplementary file 1 — Additional file 1: Figure S1. Gene Ontology enrichment analysis of genes with spliceosomal introns in the genome of M. mackini plotted in the GO semantic space using GO-Figure!. The colour scale represents the significance of the enrichmentand the size of the circles stands for the number of genes with spliceosomal introns with that particular annotation. Where available, functional categories are shown in the legend. [file 12915_2023_1635_MOESM1_ESM.png]

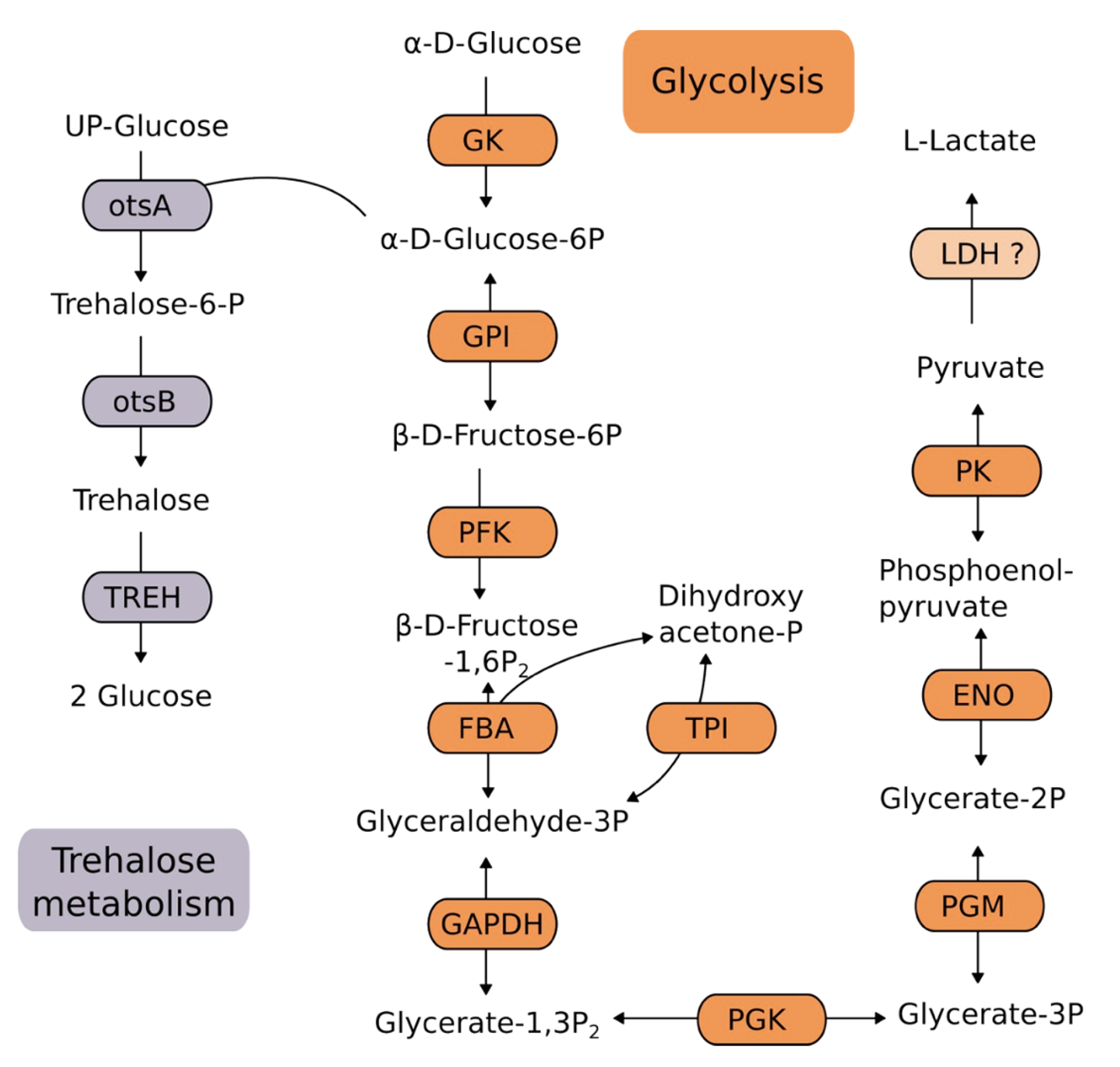

Supplement: Supplementary file 2 — Additional file 2: Figure S2. Predicted carbohydrate metabolism of M. mackini. The presence of a putative lactate dehydrogenaseenzyme has been deduced from the comparison with LDH / malate dehydrogenasehomologs. [file 12915_2023_1635_MOESM2_ESM.png]
